# Tyrosine phosphatases such as SHP-2 act in a balance with Src-family kinases in stabilization of postsynaptic clusters of acetylcholine receptors

**DOI:** 10.1186/1471-2202-8-46

**Published:** 2007-07-02

**Authors:** Alain A Camilleri, Raffaella Willmann, Gayathri Sadasivam, Shuo Lin, Markus A Rüegg, Matthias Gesemann, Christian Fuhrer

**Affiliations:** 1 Brain Research Institute, University of Zurich, Winterthurerstrasse 190, CH-8057 Zurich, Switzerland; 2 Biozentrum, University of Basel, Klingelbergstrasse 70, CH-4056 Basel, Switzerland

## Abstract

**Background:**

Development of neural networks requires that synapses are formed, eliminated and stabilized. At the neuromuscular junction (NMJ), agrin/MuSK signaling, by triggering downstream pathways, causes clustering and phosphorylation of postsynaptic acetylcholine receptors (AChRs). Postnatally, AChR aggregates are stabilized by molecular pathways that are poorly characterized. Gain or loss of function of Src-family kinases (SFKs) disassembles AChR clusters at adult NMJs *in vivo*, whereas AChR aggregates disperse rapidly upon withdrawal of agrin from cultured *src*^-/-^;*fyn*^-/- ^myotubes. This suggests that a balance between protein tyrosine phosphatases (PTPs) and protein tyrosine kinases (PTKs) such as those of the Src-family may be essential in stabilizing clusters of AChRs.

**Results:**

We have analyzed the role of PTPs in maintenance of AChR aggregates, by adding and then withdrawing agrin from cultured myotubes in the presence of PTP or PTK inhibitors and quantitating remaining AChR clusters. In wild-type myotubes, blocking PTPs with pervanadate caused enhanced disassembly of AChR clusters after agrin withdrawal. When added at the time of agrin withdrawal, SFK inhibitors destabilized AChR aggregates but concomitant addition of pervanadate rescued cluster stability. Likewise in *src*^-/-^;*fyn*^-/- ^myotubes, in which agrin-induced AChR clusters form normally but rapidly disintegrate after agrin withdrawal, pervanadate addition stabilized AChR clusters. The PTP SHP-2, known to be enriched at the NMJ, associated and colocalized with MuSK, and agrin increased this interaction. Specific SHP-2 knockdown by RNA interference reduced the stability of AChR clusters in wild-type myotubes. Similarly, knockdown of SHP-2 in adult mouse soleus muscle by electroporation of RNA interference constructs caused disassembly of pretzel-shaped AChR-rich areas *in vivo*. Finally, we found that *src*^-/-^;*fyn*^-/- ^myotubes contained elevated levels of SHP-2 protein.

**Conclusion:**

Our data are the first to show that the fine balance between PTPs and SFKs is a key aspect in stabilization of postsynaptic AChR clusters. One phosphatase that acts in this equilibrium is SHP-2. Thus, PTPs such as SHP-2 stabilize AChR clusters under normal circumstances, but when these PTPs are not balanced by SFKs, they render clusters unstable.

## Background

Neural networks are shaped through the formation, stabilization and elimination of synapses that connect neurons with their targets. The neuromuscular junction (NMJ), a model synapse in the peripheral nervous system, forms by the contact of motor neurons and muscle fibers. These interactions lead to a polyinnervated synapse at birth, in which acetylcholine receptors (AChRs) are clustered in a flat, plaque-like postsynaptic membrane. In a postnatal phase of elimination, NMJs mature and AChRs become stabilized at the crests of postjunctional folds to form pretzel-shaped areas, while all but one axon withdraw in a process in which adjacent AChRs are destabilized [1-3]. Mechanisms of stabilization of AChR clusters are thus important for proper postnatal maturation of the NMJ, which ultimately will allow for correct nerve-evoked muscle contractibility.

The molecular processes that first form NMJs are well known. Neural agrin, by activating the kinase MuSK, plays a crucial role by triggering downstream signaling pathways that cause clustering and tyrosine phosphorylation of AChRs, as reviewed recently [4,5]. Neural activity dissolves receptor clusters that are not protected by local agrin deposition in the basal lamina, thereby shaping the postsynaptic architecture [6]. In cultured myotubes, a short pulse of agrin leads to long-lasting MuSK phosphorylation and normal AChR clustering much later, implying that, once activated, a balance of downstream protein tyrosine kinases (PTKs) and protein tyrosine phosphatases (PTPs) keeps postsynaptic clustering mechanisms activated [7].

Much less is known about the molecular pathways that mature NMJs postnatally and stabilize adult pretzel-shaped AChR clusters. Although MuSK is involved [8], these pathways differ from those in NMJ induction [9,10]. Stability of AChR clusters can be modeled in cultured myotubes, by adding and then removing agrin, and studying cluster dispersal in the withdrawal phase. Despite the difference in time scale, this method reveals many parallels to postnatal stabilization of the NMJ *in vivo*, which can be assessed by electroporating interfering constructs into mouse soleus muscle [8,11]. Thus, the protein complex associated with utrophin including the components dystroglycan and dystrobrevin, and Src-family kinases (SFKs), stabilize the postsynapse and AChR clusters both *in vivo *and in cultured myotubes *in vitro *[11-16]. SFKs are activated by agrin [17], interact with AChRs [18,19], and maintain AChR β subunit phosphorylation and interaction of the receptor with its anchoring protein rapsyn [11]. In cultured *src*^-/-^;*fyn*^-/- ^myotubes, agrin or laminin induce normal AChR clustering, but the clusters dissemble rapidly within a few hours after withdrawal of these factors [12,14]. SFKs act in a dual mechanism with cholesterol-rich lipid microdomains related to lipid rafts, as SFKs promote normal microdomain assembly, while the microdomains allow SFKs to act upon postsynaptic proteins [20]. *In vivo*, interfering with SFK function causes postsynaptic disintegration of adult NMJs. Interestingly, decreasing or increasing SFK activity through expression of dominant-negative (kinase-dead) or constitutively active Src both cause disassembly of AChRs aggregates *in vivo *[11]. This raises the possibility that balanced SFK activity is important for postsynaptic stability and suggests that PTPs may be involved in stabilization of AChR clusters as well. One PTP known to be enriched at the NMJ is the non-receptor phosphatase SHP-2 [21]. SHP-2 can modulate agrin/MuSK signaling and neuregulin-regulated AChR transcription [21,22], making it a good candidate for postsynaptic signaling processes.

We have therefore analyzed the role of PTPs, especially SHP-2, in the stabilization of AChR clusters, and their interplay with SFKs. We find that phosphatases such as SHP-2 stabilize AChR clusters after withdrawal of agrin from cultured myotubes. SHP-2 is also necessary to stabilize adult AChR pretzels at NMJs in mouse soleus muscle *in vivo*. Interestingly, in *src*^-/-^;*fyn*^-/- ^myotubes SHP-2 is overexpressed, and blocking PTP activity restores AChR cluster stability completely, showing that a fine balance of PTPs with SFKs is crucial for maintaining AChR clusters.

## Results

### PTP inhibition by pervanadate reduces the stability of agrin-induced AChR clusters

**Figure 1:**
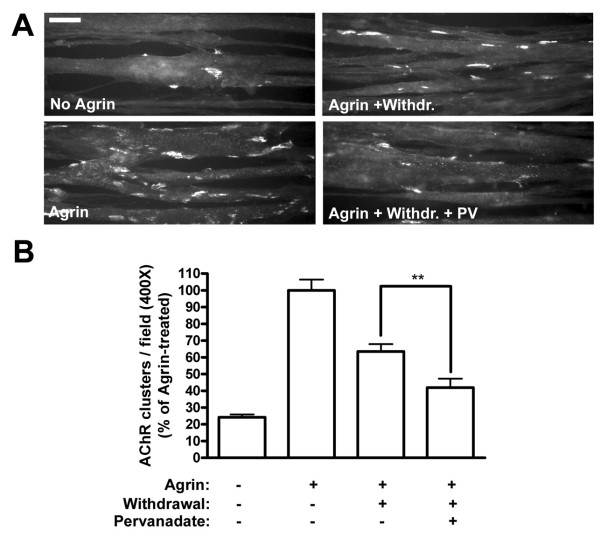
**PTP inhibition by pervanadate reduces the stability of agrin-induced AChR clusters in C2C12 myotubes**. (A) Myotubes were treated with agrin (1 nM) for 6–8 h, after which PTPs were blocked with 20 μM pervanadate at the point of agrin withdrawal (Agrin + Withdr. + PV) for a withdrawal period of 16 h. In controls, cultures were left untreated (No Agrin), treated with agrin alone for 6–8 h (Agrin), or treated with agrin followed by withdrawal for 16 h (Agrin + Withdr.). Cells were also treated with agrin for 6–8 h + 16 h, and this produced the same amount of clusters as a 6–8 h agrin incubation (data not shown). AChRs were stained with rhodamine-α-BTX and analyzed by fluorescence microscopy. Scale bar, 40 μm. (B) The number of AChR clusters per field was calculated using ImageJ software, using fixed intensity thresholds (150–255) and minimum area of 100 pixels occupied by a cluster. The number of AChR clusters per field (400× magnification) is shown as the percentage of clusters in agrin-treated cells (Agrin) (means ± SEM, *N *= 50 from four similar experiments). Phosphatase inhibition significantly decreases the number of pre-existing, agrin-induced AChR clusters following agrin removal (** *p *< 0.01; two-tailed paired *t *test).

To address the role of the activity of PTPs in the stabilization of agrin-induced AChR clusters, we made use of the potent PTP inhibitor sodium pervanadate [23,24] and cultured C2C12 myotubes, in which AChRs were visualized by treatment with fluorescent α-bungarotoxin (α-BTX). PTP inhibition did not lead to any change in the number of spontaneous AChR clusters observed per field when compared to the untreated control (data not shown). The stability of AChR clusters can be modeled in cell culture by incubating C2C12 myotubes with agrin to induce maximal clustering, followed by agrin withdrawal, washing the cells, incubating them in agrin-free medium for a number of hours, and counting clusters at the end of this withdrawal period [7,11,12,14]. When pervanadate was added at the time of agrin withdrawal, significantly less AChR clusters were observed after a 16 h withdrawal period than in controls lacking pervanadate (Figure 1). These data show that block of PTP activity enhances the disintegration of pre-existing agrin-induced AChR clusters and that therefore, PTPs are required to stabilize clusters of AChRs. This is a specific process operating in cells that were previously exposed to agrin, since block of PTP activity as such does not alter the level of spontaneous AChR clustering.

### A balance of PTP and SFK activities stabilizes AChR clusters: the instability of AChR clusters in the absence of SFKs is rescued by pervanadate treatment

**Figure 2:**
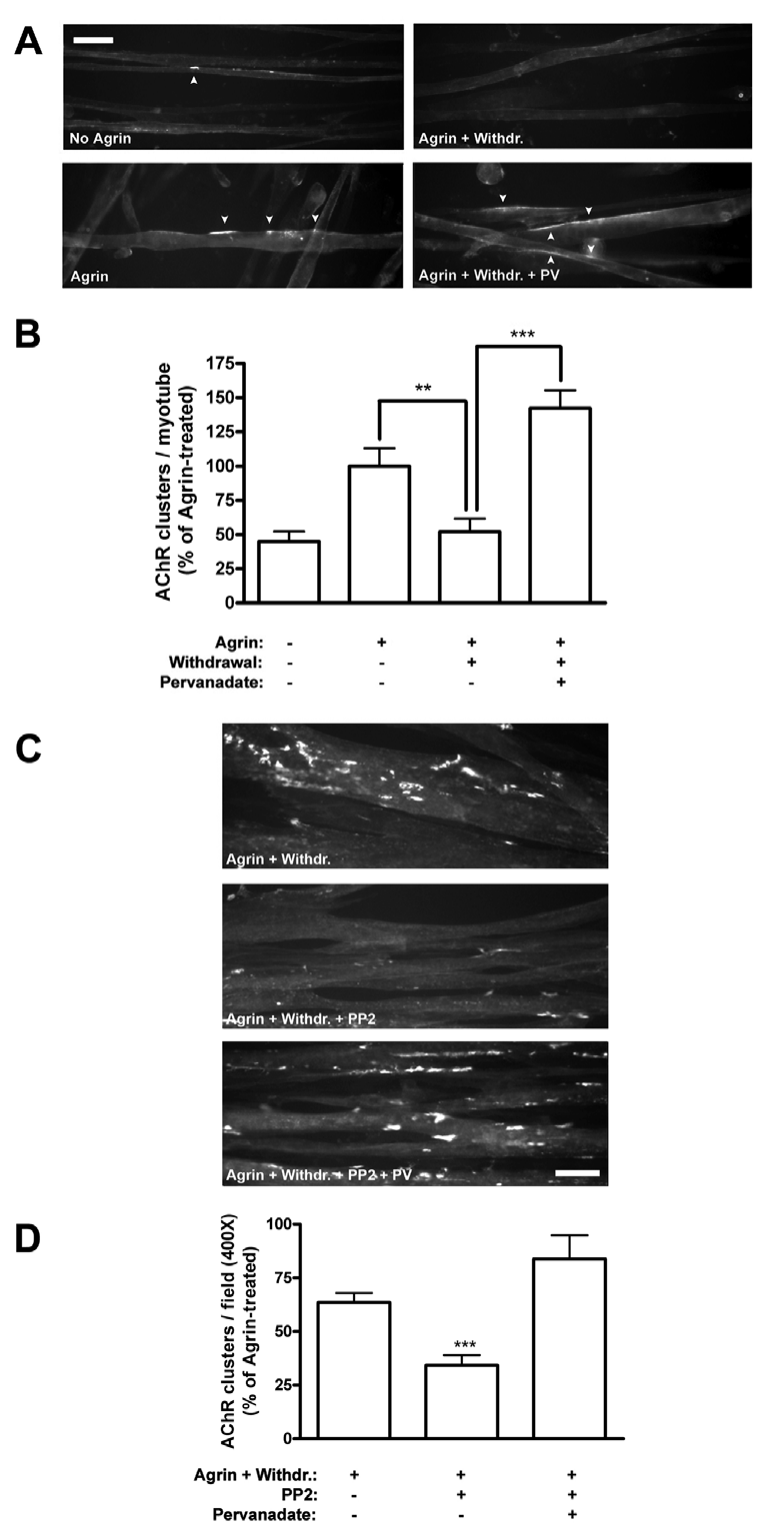
**AChR cluster stability is rescued by PTP inhibition in *src*^-/-^;*fyn*^-/- ^myotubes and PP2-treated C2C12 myotubes**. (A) PTPs were blocked with 20 μM pervanadate in *src*^-/-^;*fyn*^-/- ^myotube cultures at the point of agrin withdrawal for a period of 5 h (Agrin + Withdr. + PV), following agrin treatment for 16 h. As controls, myotubes were either left untreated (No Agrin), treated with agrin for 16 h (Agrin) or treated with agrin followed by withdrawal for 5 h (Agrin + Withdr.). AChRs were stained with rhodamine-α-BTX and analyzed by fluorescence microscopy. Scale bar, 40 μm. White arrowheads indicate AChR clusters. (B) The number of AChR clusters per myotube is shown as the percentage of clusters in agrin-treated *src*^-/-^;*fyn*^-/- ^cells (Agrin) (means ± SEM; *N *= 15 from three similar experiments; **p < 0.01, ***p < 0.001 by two-tailed paired *t *test). Pervanadate restores cluster stability following agrin withdrawal in *src*^-/-^;*fyn*^-/- ^myotubes. (C) The inhibitor PP2 was used to block SFK activity in C2C12 myotubes. Myotubes were treated with agrin for 6–8 h in the presence of 10 μM PP2 for the last 2 h (these 2 h were chosen to ensure that PP2 was effective at the point of agrin withdrawal). Agrin was removed and myotubes incubated with agrin-free medium in the presence of pervanadate and PP2 for 16 h (Agrin + Withdr. + PP2 + PV). In controls, cultures were treated with agrin followed by withdrawal for 16 h in the presence or absence of PP2 (PP2 was added 2 h before agrin removal) (Agrin + Withdr.; Agrin + Withdr. + PP2). AChRs were stained with rhodamine-α-BTX, analyzed by fluorescence microscopy. Scale bar, 40 μm. (D) The number of AChR clusters per field (400× magnification) was calculated, as described in Figure 1, as the percentage of agrin-treated C2C12 cells (Agrin) (not shown) (means ± SEM, *N *= 30 from three experiments). *** indicates significant difference to the other two bars (*p *< 0.001; two-tailed paired *t *test). Blocking SFKs with PP2 reduces the stability of pre-existing clusters in C2C12 myotubes, and pervanadate restores the stability to normal levels.

SFKs are key in stabilizing AChR clusters *in vivo *and in cultured myotubes *in vitro *[11,12,14]. In *src*^-/-^;*fyn*^-/- ^myotubes, agrin induces normal AChR clustering, but the clusters disassemble rapidly within few hours upon agrin withdrawal [14]. We analyzed whether under these circumstances PTPs may also influence the stability of AChR clusters. For this purpose we treated *src*^-/-^;*fyn*^-/- ^myotubes with agrin to induce maximal clustering, then withdrew agrin and added pervanadate at the same time, for a withdrawal period of 5 h. This pervanadate treatment prevented AChR clusters from disappearing, such that clusters remained completely stable during the withdrawal period (Figure 2A, B). In sharp contrast, omitting pervanadate led to disassembly of the agrin-induced clusters down to spontaneous levels (Figure 2A, B). Thus, block of PTP activity causes stabilization of AChR clusters in the absence of Src and Fyn. This is a specific process that only affects pre-existing agrin-induced clusters, because pervanadate treatment alone did not alter the levels of spontaneous clusters (data not shown). Moreover, when added together with agrin, pervanadate did not affect the extent of agrin-induced AChR clustering (data not shown).

Src and Fyn are not the only members of this family of kinases present in myotubes. A third member of the SFKs, Yes, is present in muscle and upregulated in *src*^-/-^;*fyn*^-/- ^myotubes [14]. This led us to investigate whether the observed destabilizing effect of PTPs was solely due to the absence of Src and Fyn, or whether this effect would also occur when all members of the SFKs in muscle were blocked. We therefore made use of the specific inhibitor PP2 to block SFKs in C2C12 myotubes [25]. We again induced AChR clustering by agrin and then removed agrin to analyze the effect on the stability of these AChR clusters. We found that when added together with agrin, PP2 had no effect on the level of clustering induced by agrin (data not shown), as has been previously observed [14]. However, upon removal of agrin, blocking SFKs with PP2 led to a two-fold decrease in the number of remaining AChR clusters, almost down to spontaneous levels (Figure 2C, D). This instability was completely prevented when PTPs were inhibited with pervanadate in the withdrawal phase (Figure 2C, D). These data confirm those from *src*^-/-^;*fyn*^-/- ^myotubes and show, firstly, that SFK activity maintains the stability of AChR clusters in cultured wild-type myotubes, similar to recent findings in muscle *in vivo *[11]. Secondly, PTPs destabilize AChR clusters in the absence of SFK activity and could indeed be the key destabilizing factor for agrin-induced AChR clusters in the absence of SFKs. Taken together, these data show that the stability of agrin-induced AChR clusters following the withdrawal of agrin requires a fine balance between the kinase activities of SFKs and the phosphatase activities of PTPs.

### Protein tyrosine phosphatase SHP-2 increasingly associates with MuSK upon agrin stimulation

We wanted to identify possible PTPs that play a role in stabilization of AChR clusters. An example of how the activity of a PTP is required in positively controlling the actions of a kinase is the regulation of Src activity by the SH2 domain-containing protein tyrosine phosphatase SHP-2 [26]. SHP-2 is enriched at neuromuscular synapses and colocalizes with AChRs *in vivo *[21]. In cultured myotubes, SHP-2 is a major PTP and can control the phosphorylation level of MuSK and the extent of agrin-induced AChR clustering [22]. SHP-2 is therefore a likely candidate PTP for playing roles in the stabilization process of AChR clusters.

**Figure 3:**
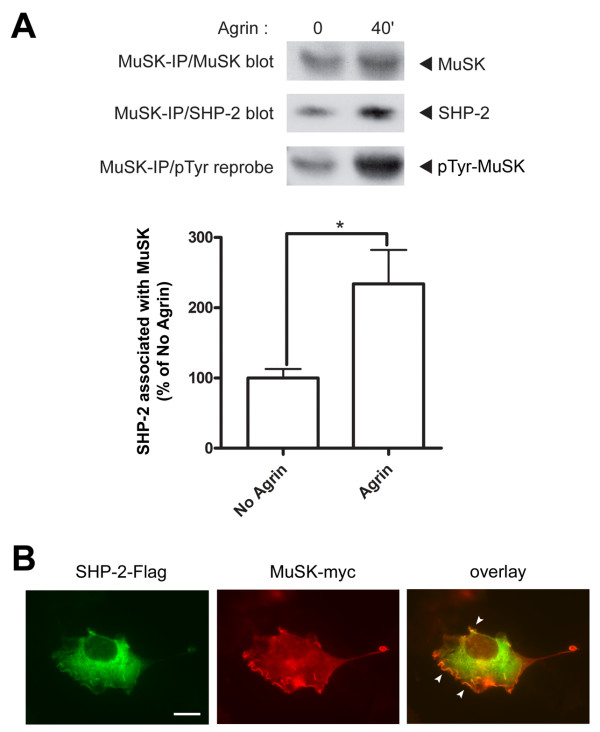
**Protein tyrosine phosphatase SHP-2 increasingly associates with MuSK upon agrin stimulation**. (A) C2C12 myotubes were treated with 0.5 nM agrin for 40 min. Following MuSK immunoprecipitation, immunoblotting for SHP-2 and MuSK was carried out, followed by reprobing for phosphorylation of MuSK. Levels of SHP-2 co-precipitated with MuSK were quantitated (normalized for the amount of precipitated MuSK) as percentage of No Agrin control. Co-immunoprecipitation of SHP-2 with MuSK indicates its specific association with MuSK, which increases significantly upon agrin-induced MuSK phosphorylation (means ± SEM, from seven experiments; * *p *< 0.05; two-tailed unpaired *t *test). As a control, the MuSK antibody was omitted in the immunoprecipitation, leading to no substantial signal in the Western blots (not shown). (B)COS cells were co-transfected with SHP-2-Flag and MuSK-myc constructs. Cells were then fixed and stained with anti-Flag/Alexa488 and anti-myc/Alexa546 antibodies. SHP-2 localizes throughout the whole of the cytoplasm, while MuSK localization is concentrated along the plasma membrane. White arrowheads in the overlay indicate a colocalization of MuSK and SHP-2 along membrane ruffles on the plasma membrane. Scale bar, 40 μm.

We first determined whether SHP-2 is in a position to affect cluster stabilization by testing its association with MuSK. We treated C2C12 myotubes with agrin to induce MuSK phosphorylation, following which we immunoprecipitated MuSK and probed for both MuSK and SHP-2. We found that SHP-2 is co-immunoprecipitated together with MuSK, also without agrin treatment, and that the association between the two proteins increases 2.3-fold following MuSK phosphorylation by agrin (Figure 3A).

Since SHP-2 is known to colocalize with AChRs and hence presumably also with MuSK at NMJs [21], we investigated this association between MuSK and SHP-2 further using a heterologous cell system. We transfected COS cells with *myc*-tagged MuSK and *Flag*-tagged SHP-2 constructs, and visualized MuSK and SHP-2 in the transfected cells by immunocytochemistry. As expected, being a ubiquitously-expressed non-receptor PTP, SHP-2 was localized throughout the whole of the cell, but also along membrane ruffles on the plasma membrane (Figure 3B). On the other hand, MuSK showed more of a membrane localization, characteristic for a receptor tyrosine kinase (RTK). SHP-2 and MuSK colocalized within these membrane ruffles present along the plasma membrane (Figure 3B). They however did not cause the relocalization of one another, since singly-transfected COS cells also exhibited similar distribution patterns of the expressed proteins (data not shown).

From these results we can conclude that MuSK and SHP-2 colocalize in ruffles along the plasma membrane. They also associate with one another, and SHP-2 increases its association to MuSK upon MuSK activation by agrin, perhaps through an interaction of the SH2 domains in SHP-2 with MuSK phosphorylation sites. The association with MuSK highlights that SHP-2 is in a position to affect AChR cluster stability.

### Knockdown of SHP-2 in myotubes by vector-driven shRNA specifically reduces SHP-2 protein levels

**Figure 4:**
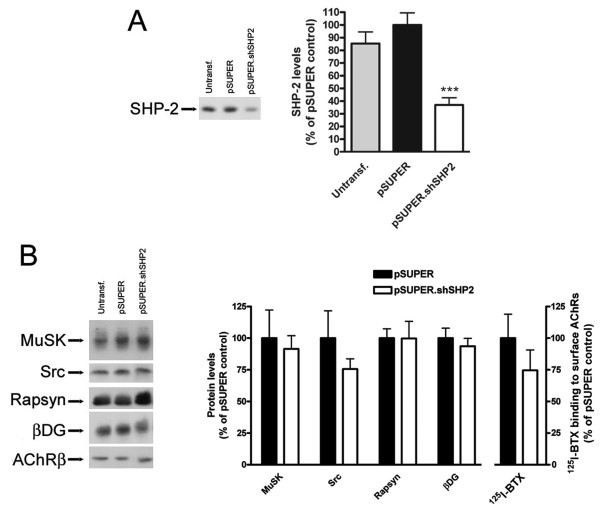
**Gene-specific targeting of SHP-2 in C2C12 myotubes through vector-driven shRNA specifically knocks down SHP-2 protein**. (A) C2C12 myotubes were transfected with *pSUPER.shSHP2 *construct targeting a specific region of the murine SHP-2 ORF. No transfection (Untransf.) or *pSUPER *vector only (pSUPER) transfected myotubes served as controls. Western blot analysis of SHP-2 levels revealed that *pSUPER.shSHP2 *knocked down > 60% of endogenous SHP-2. SHP-2 levels were quantitated by densitometric scanning and are shown as % of *pSUPER *vector only transfected myotubes (means ± SEM, from eight experiments). *** indicates a significant difference to the other bars (*p *< 0.0001; two-tailed unpaired *t *test). (B) The effect of SHP-2 gene targeting on levels of several postsynaptic proteins and functional surface AChRs was analyzed by *pSUPER.shSHP2 *transfection followed by Western blot analysis or radioligand binding assays (^125^I-α-BTX binding to intact C2C12 myotubes). This revealed that SHP-2 knockdown does not significantly influence the levels of MuSK, Src, rapsyn and β-dystroglycan. Protein levels were calculated and shown as % of respective *pSUPER *vector only transfected myotubes (means ± SEM, from eight experiments). Equal amounts of overall protein were loaded on the gels. Similarly, ^125^I-α-BTX binding to surface AChRs in intact C2C12 myotubes shows that following SHP-2 knockdown there is no significant effect on the surface levels of AChRs, when compared to radioligand binding on control transfected myotubes.

**Figure 5:**
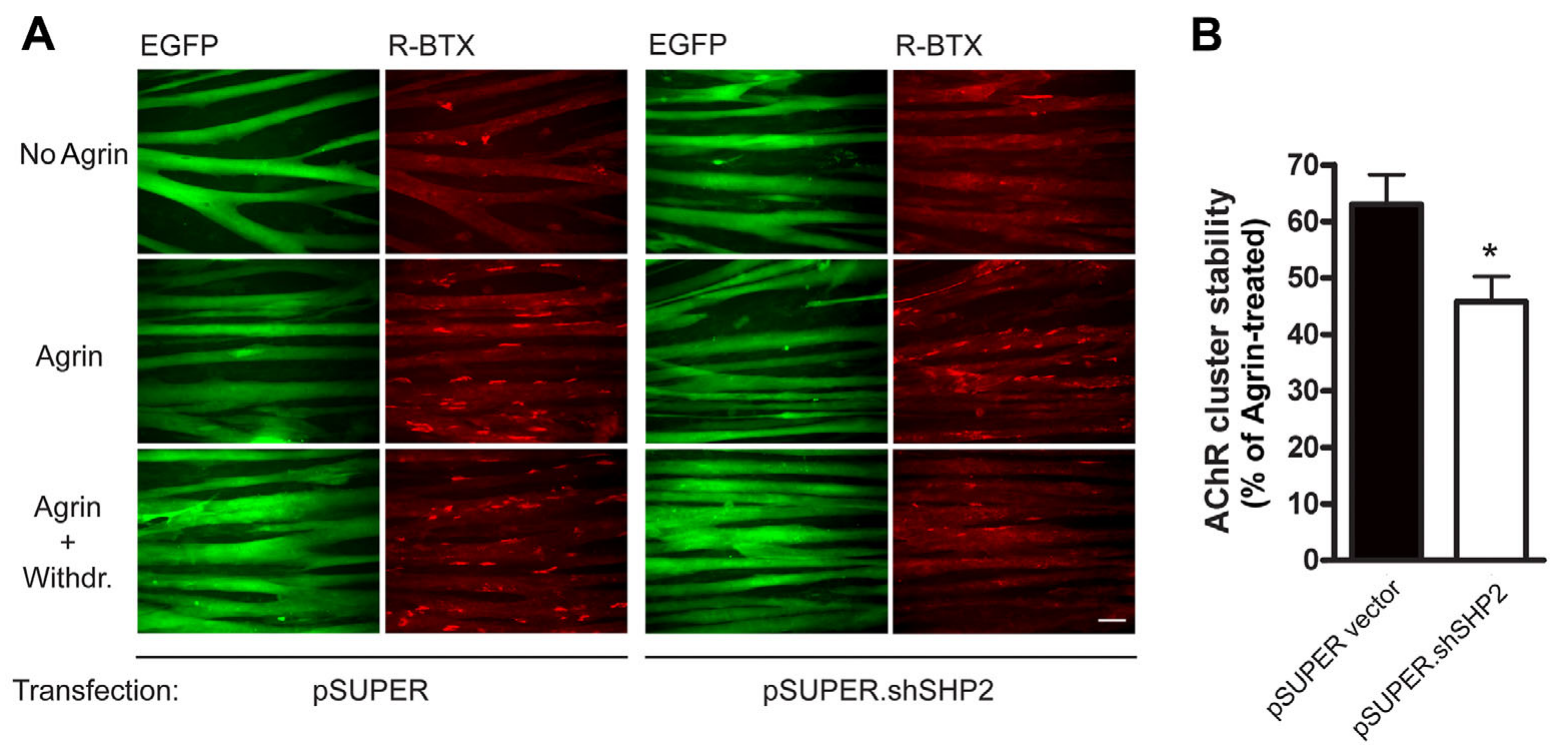
**Upon knockdown of SHP-2 in C2C12 myotubes, AChR clusters are less stable**. (A) *pSUPER*-, and *pSUPER.shSHP2*-transfected C2C12 myotubes were either left untreated (No Agrin), treated with agrin for 16 h (Agrin), or treated with agrin (16 h) followed by withdrawal of agrin for 8 h (Agrin+Withdr.). AChRs were identified by rhodamine-α-BTX staining and cells were analyzed by fluorescence microscopy. Scale bar, 40 μm. Transfected myotubes expressing EGFP indicated that transfection efficiency at the myotube level approached 100%. The data show that agrin induces AChR clustering both in *pSUPER*- and *pSUPER.shSHP2*-transfected myotubes. After the withdrawal phase, less clusters remain in the case of SHP-2 knockdown when compared to the control. (B) The stability of clusters was quantitated by setting the number of AChR clusters in agrin-treated myotubes (Agrin) to 100%, both for *pSUPER*-, and *pSUPER.shSHP2*-transfected myotubes. Clusters after the withdrawal period (Agrin + Withdr.) were then calculated accordingly. The absence of SHP-2 renders AChR clusters significantly less stable when compared to control transfected myotubes (means ± SEM, *N *= 30 from four similar experiments; *p < 0.05; two-tailed unpaired *t *test).

To address the role of SHP-2 in stabilization of AChR clusters, we used a knockdown approach by RNA interference (RNAi). We cloned three short-hairpin RNA (shRNA) loops into the *pSUPER.gfp *vector and tested their efficacy in downregulating SHP-2 expression in C2C12 myotubes. We used shRNA loops generated against different regions of the murine SHP-2 open-reading frame (ORF), based on already published and successfully used siRNA or shRNA sequences [27,28]. These constructs co-expressed EGFP, allowing easy identification of the successfully transfected myoblasts, and observation of the differentiation of these myoblasts into mature myotubes. Transfection efficiencies at the myoblast level were 40–50%, which upon differentiation and fusion led to close to 100% of the myotubes expressing EGFP (see Figure 5A). By Western blot analysis of C2C12 myotube lysates we probed for the effective suppression of endogenous SHP-2 protein. One of the three constructs, termed *pSUPER.shSHP2*, led to a strong (> 60%) reduction of endogenous SHP-2 protein levels when compared to control transfected myotubes (*pSUPER *vector only; Figure 4A). We therefore proceeded to analyze the effect of SHP-2 downregulation on other postsynaptic proteins. In immunoblots, levels of other postsynaptic proteins such as MuSK, Src, rapsyn and β-dystroglycan were not affected by SHP-2 knockdown (Figure 4B). Using radioligand binding of ^125^I-α-BTX we probed functional surface AChRs following transfection with *pSUPER.shSHP2 *and observed no significant effect (Figure 4B). *pSUPER.shSHP2 *thus allows specific and strong knockdown of SHP-2 without interfering with other muscle proteins.

### SHP-2 is required for stabilization of AChR clusters in cultured myotubes

To study the role of SHP-2 for AChR cluster stability, we used C2C12 myotubes and suppressed SHP-2 by shRNA as described above. We incubated the cells with agrin to induce maximal clustering and withdrew the agrin to assess the stability of the clusters. In both control cells (*pSUPER*) and after SHP-2 knockdown (*pSUPER.shSHP2*), spontaneous AChR aggregates were present and agrin induced strong clustering of the receptors (Figure 5A), similar to a recent report [22]. After withdrawal of agrin for 8 h, few clusters remained in *pSUPER.shSHP2*-transfected cells whereas more clusters were present in *pSUPER*-treated cells (Figure 5A).

To quantitate the effect of SHP-2 knockdown on the stability of agrin-induced AChR clusters, we normalized the number of AChR clusters following agrin withdrawal ("Agrin + Withdr.") to the number of clusters that were induced by agrin ("Agrin") in *pSUPER.shSHP2*- and *pSUPER*-transfected cells. The result shows that there is a significant decrease in the stability of AChR clusters following agrin withdrawal when SHP-2 expression is knocked down by shRNA (Figure 5B). These data illustrate that SHP-2 is required for optimal stabilization of AChR clusters in cultured myotubes.

### Requirement of SHP-2 for stabilization of postsynaptic AChR pretzels at the NMJ *in vivo*

To address the role of SHP-2 in stabilization of postsynaptic AChRs at the NMJ *in vivo*, we used RNA interference in soleus muscles of adult mice as described earlier [8,11]. The shRNA construct *pSUPER.shSHP2*, together with a construct expressing GFP containing a nuclear localization signal (NLS-GFP), was introduced into adult soleus muscle by electroporation. After 6 weeks, muscles were dissected, stained as whole mount preparations with rhodamine-α-BTX and antibodies against neurofilament and synaptophysin, and analyzed by confocal microscopy including 3D reconstruction.

**Figure 6:**
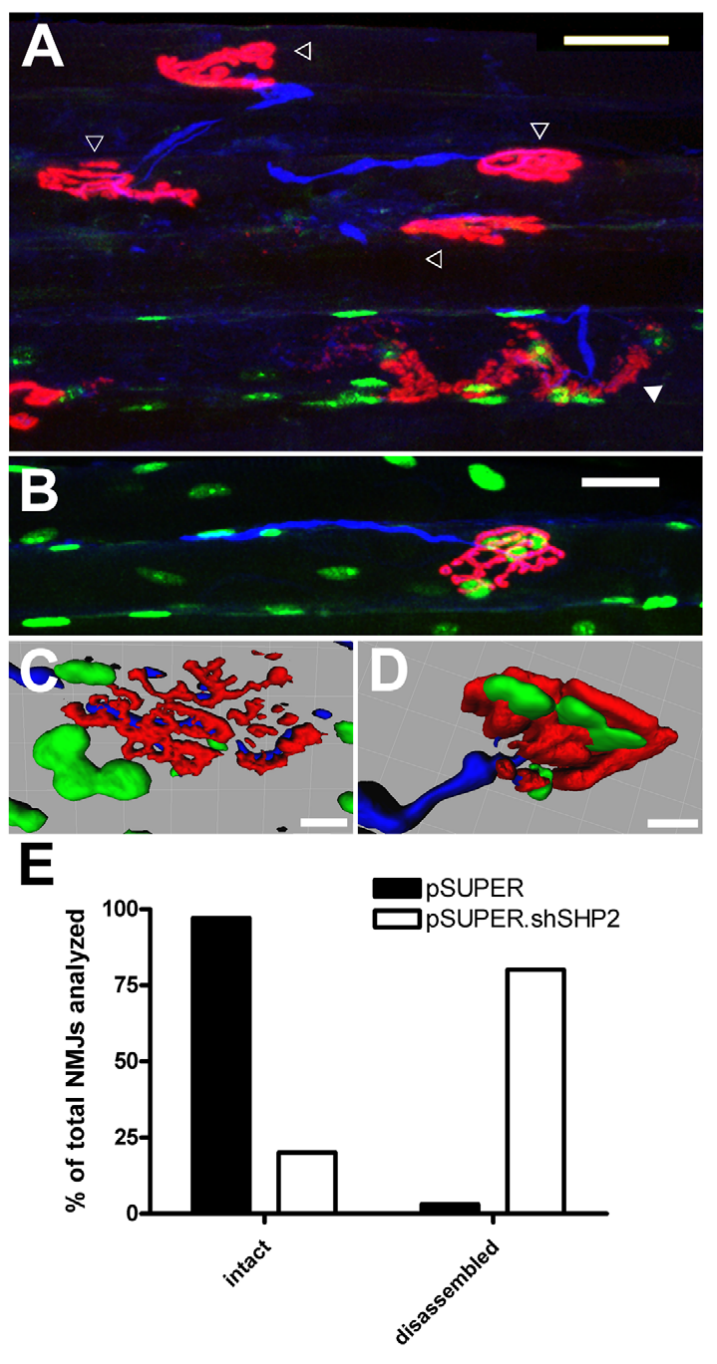
**Electroporation of SHP-2 shRNA construct into soleus muscle of adult mice leads to disassembly of NMJs**. Adult mice soleus muscles were electroporated *in vivo *with a mixture of *pSUPER.shSHP2 *and NLS-GFP constructs (A, C), or *pSUPER *vector and NLS-GFP as control (B, D). After six weeks muscles were dissected, whole mounts of fibers prepared and stained with rhodamine-α-BTX and a mixture of neurofilament and synaptophysin antibodies followed by Cy5 secondary antibody. NMJs were analyzed by confocal microscopy, whereby successfully electroporated muscle fibers were identified by the expression of NLS-GFP in myonuclei. (A) Expression of *pSUPER.shSHP2 *results in NMJ disassembly in GFP-positive myofibers (closed arrowhead). NMJs of GFP-negative fibers remain intact (open arrowheads). (B) Expression of control *pSUPER *vector and NLS-GFP has no effect on the NMJs. A three-dimensional reconstruction of a confocal image of a muscle electroporated with *pSUPER.shSHP2 *and NLS-GFP (C), or control *pSUPER *vector and NLS-GFP (D) shows details of the NMJs. (C) A 3D view from inside the muscle illustrates that expression of SHP-2 shRNA leads to disassembly of the NMJ, loss of the usual morphology and pretzel shape, and a resulting fragmented appearance of the NMJ, with the nerve becoming visible through the pretzel remnants. (D) In control electroporated muscle fibers, GFP-positive synaptic nuclei are located below the intact pretzel-shaped accumulations of AChRs and no nerve is visible through the pretzel. (E) The number of intact and disassembled NMJs is shown for control (*pSUPER*) vector and *pSUPER.shSHP2 *electroporated muscle fibers, as percentage of the total number of NMJs analyzed (total of 25 endplates from 3 mice for *pSUPER *and 15 endplates from 5 mice for *pSUPER.shSHP2*). Only endplates with synapse-associated nuclei expressing NLS-GFP were analyzed. Scale bars, 30 μm in A, B; 10 μm in C, D.

The *in vivo *expression of *pSUPER.shSHP2 *led to the disassembly of adult pretzel-shaped AChR clusters (Figure 6A, closed arrowhead). This was in sharp contrast to the intact AChR pretzel structures observed at NMJs of neighboring GFP-negative myofibers (Figure 6A, open arrowheads) and of myofibers of mice electroporated with control *pSUPER *vector and NLS-GFP (Figure 6B).

Disassembled NMJs of myofibers expressing SHP-2 shRNA lost their typical pretzel morphology and were fragmented (example in Figure 6C) and sometimes altogether widened in appearance (example in Figure 6A). In addition, the synapse-associated myonuclei were sometimes no longer tightly packed subsynaptically but associated more loosely with the disassembled AChR pretzel (Figure 6C). In contrast, in myofibers electroporated with control *pSUPER *vector, the nerve was hardly visible from inside the muscle and synaptic nuclei were clustered just beneath the AChR pretzels (Figure 6D).

A quantitative assessment was made following the criteria described earlier [11](see also Method section), by analyzing many NMJs from several mice, for both *pSUPER.shSHP2 *and *pSUPER *control vector. In the control, almost all NMJs scored as intact, but in myofibers where SHP-2 was suppressed, only a minority (less than 25%) of all NMJs analyzed was intact and most synapses scored as disassembled. These data show that SHP-2 is required for maintenance of the adult postsynaptic apparatus of the NMJ *in vivo *including typical pretzel-shaped AChR domains and synaptic myonuclei. These results are very similar to the role of SHP-2 in stabilization of agrin-induced AChR clusters in myotubes *in vitro*.

### Elevated levels of SHP-2 in the absence of Src and Fyn

**Figure 7:**
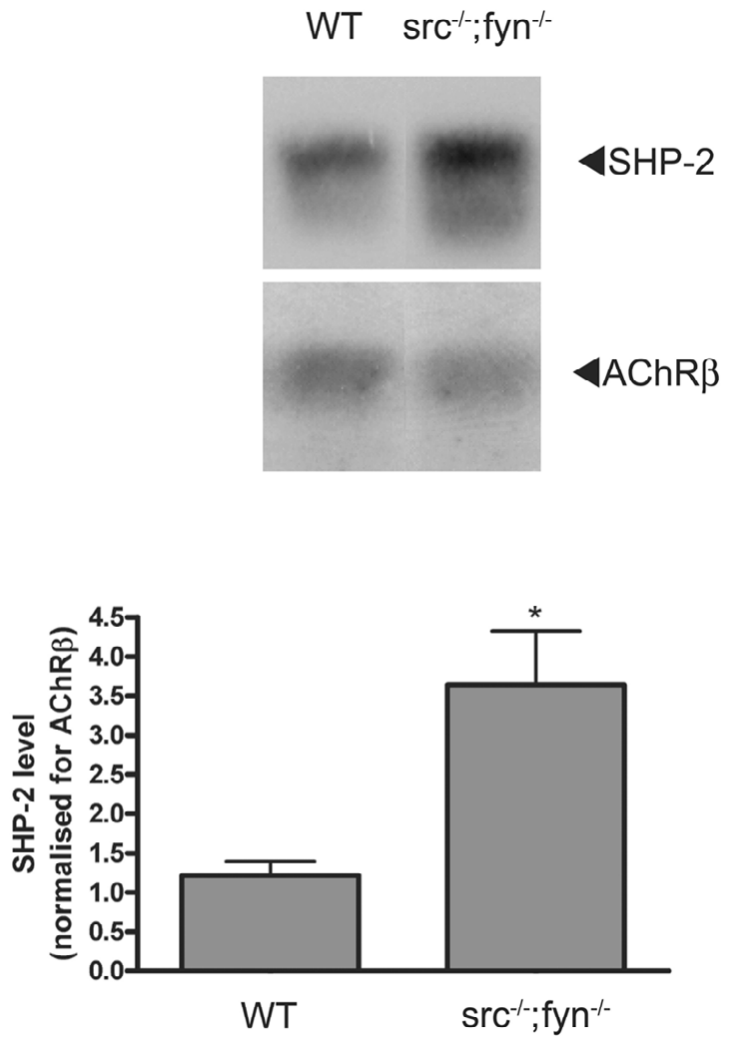
**Elevated protein levels of SHP-2 in the absence of Src and Fyn**. Western blot analysis of total SHP-2 protein levels in wild-type versus *src*^-/-^;*fyn*^-/- ^myotubes reveals that in the absence of Src and Fyn there is a >3-fold increase in the protein levels of SHP-2. SHP-2 levels are shown for both wild-type and *src*^-/-^;*fyn*^-/- ^myotubes; equal amounts of cellular protein were loaded on the gel. In the quantitation, SHP-2 levels are normalized for the level of AChR β subunit (*p < 0.05; two-tailed unpaired *t *test).

We have observed similar destabilizing effects on AChR clusters in C2C12 myotubes upon PTP inhibition by pervanadate, and upon knockdown of SHP-2 by shRNA (Figures 1 and 5). This instability is also present in the absence of Src and Fyn [14], or upon blocking SFKs with the inhibitor PP2, and is rescued completely by inhibition of phosphatase activity by pervanadate (Figure 2). These observations lead to the conclusion that it is the balance between PTK and PTP activity that regulates the stability of AChR clusters in myotubes. For this reason we sought to compare the levels of SHP-2 between *src*^-/-^;*fyn*^-/- ^myotubes and wild-type myotubes. We carried out Western blot analysis of myotube lysates, and found that SHP-2 levels are 3-fold higher in myotubes lacking Src and Fyn compared to wild-type myotubes (Figure 7). This finding suggests that these high levels of SHP-2 may cause cluster destabilization and further strengthens our proposal that a crucial aspect for AChR cluster stabilization is the balance between Src and Fyn, and PTPs such as SHP-2.

## Discussion

We have analyzed the role of PTPs in the stabilization of AChR clusters and report that PTPs are required for cluster maintenance and that the fine balance between PTPs and SFKs is a key aspect in this process. On one hand, block of PTPs by pervanadate or knockdown of SHP-2 in wild-type myotubes both rendered agrin-induced clusters less stable. *In vivo*, SHP-2 knockdown in muscle caused disassembly of adult pretzel-shaped AChR-rich areas at NMJs. On the other hand, pervanadate restored the defective stability of clusters in myotubes lacking SFK activity, and *src*^-/-^;*fyn*^-/- ^myotubes had elevated levels of SHP-2. Thus, PTPs such as SHP-2 stabilize AChR clusters under normal circumstances, but when these PTPs are not balanced by SFKs, they render clusters unstable.

### PTPs such as SHP-2 stabilize AChR clusters

We used two approaches to illustrate the role of PTPs in the maintenance of AChR clusters: inhibiting the activity of all PTPs by pervanadate, and testing the role of a candidate PTP, SHP-2. The stability of AChR clusters was modeled in cell culture, by adding and then removing agrin, and pervanadate was added at the point of agrin withdrawal to specifically test its effect on the maintenance of pre-existing agrin-induced clusters. The withdrawal and washing procedure removes the vast majority of agrin from cells, such that signaling processes in the myotubes during the withdrawal phase do not reflect the activity of extracellular agrin but ongoing processes within the myotubes [7]. When agrin is withdrawn from wild-type myotubes, AChR clusters slowly disintegrate over the course of several hours [29]. Block of PTPs by pervanadate accelerated the decay of clusters, showing that PTP activity is a requirement for optimal AChR cluster stability. This was a specific process, since pervanadate as such did not alter levels of spontaneous AChR clustering. Rather, following an agrin stimulation to produce maximal aggregates, PTPs act to fully maintain these clusters.

PTPs exist in many families, as receptor tyrosine phosphatases or cytosolic non-receptor phosphatases, similar to kinase families. While several PTPs could share the function of maintaining AChR clusters, we identified SHP-2 as one PTP that plays such a role. SHP-2 has previously been put into the context of the NMJ as a regulator of AChR synthesis and MuSK activity [21], and we first further verified whether SHP-2 is located to play a postsynaptic role. We found SHP-2 to associate with MuSK, and this association to be increased (2.3-fold) by an agrin treatment that caused heavy MuSK tyrosine phosphorylation. In COS cells, MuSK and SHP-2 showed colocalization in membrane ruffles at the plasma membrane, implying that they can interact with each other independently of muscle-specific linker proteins. These data show that SHP-2, by increasingly interacting with MuSK, is positioned to control postsynaptic stability of AChR clusters.

We determined the role of SHP-2 in the stabilization of AChR clusters by an RNAi strategy, using vector-driven shRNA and short-hairpin loops that were previously used to knock down SHP-2 [27,28]. One of the shRNA constructs tested (*pSUPER.shSHP2*) was effective in myotubes when using an efficient transfection protocol that allowed its expression in nearly 100% of myotubes. *pSUPER.shSHP2 *massively reduced protein amounts of SHP-2 without significantly interfering with other postsynaptic proteins such as MuSK, Src, rapsyn and β-dystroglycan or with levels of functional AChR at the cell surface. This specificity in knockdown allowed the study of the role of SHP-2 in AChR cluster stability.

In the absence of normal SHP-2 levels, spontaneous AChR aggregates were present and AChR clusters were induced by agrin, but the stability of these clusters was reduced compared to controls, showing that, similar to the results described above with pervanadate, SHP-2 stabilizes AChR clusters. SHP-2 was also important at NMJs *in vivo*, because electroporation of *pSUPER.shSHP2 *into adult mouse soleus muscle led to disassembly of the postsynapse: pretzel-shaped AChR-rich areas became fragmented and some lost their overall pretzel outline, and sometimes myonuclei were no longer packed subsynaptically but more loosely associated with fragmented AChR cluster remnants. Taken together, our data show that SHP-2 is a PTP that, perhaps together with other PTPs, contributes to the maintenance of AChR clusters at NMJs *in vivo *and in cultured myotubes *in vitro*.

Upon knockdown of SHP-2, fragments of AChR clusters were visible at NMJs but not in cultured myotubes from which agrin had been withdrawn. In myotubes, cluster disappearance appears as a statistical process whereby some clusters still exist while others have vanished and are outside the detectable ranges used in our method. This could point towards differences in cluster dynamics between our *in vitro *and *in vivo *systems. Such differences could result from one or more of several possibilities: at NMJs, the turnover of AChRs is much slower and the subsynaptic cytoskeleton more elaborate, presumably immobilizing the AChR in a stronger fashion; cultured myotubes lack innervation and the continuous presence of basal lamina-anchored agrin as seen *in vivo*; and the timing of AChR cluster disassembly is different as indicated by the different time scale of our *in vivo *vs. *in vitro *analysis. However, equally likely is the possibility that upon SHP-2 knockdown and agrin withdrawal, AChR cluster fragments do exist in cultured myotubes but cannot be visualized due to low intensity; in comparison the density of AChRs in pretzels is much higher *in vivo*. Furthermore, fragments of AChR pretzels may completely disappear from *pSUPER.shSHP2*-expressing myofibers after prolongated times.

There are several possibilities of how SHP-2 could act to maintain clusters. The agrin-induced increase in MuSK-SHP-2 interaction is very similar to SHP-2 association with tyrosine phosphorylated DOK1, a member of insulin receptor substrate protein family that binds β3. Association with DOK1 also occurs under basal conditions, and upon stimulation of DOK1 by insulin-like growth factor I (IGI-I), SHP-2-DOK1 association increases 2.7-fold. This association is important for DOK1 to present SHP-2 to downstream SHPS-1 [30,31]. In a similar fashion, SHP-2 associates, via its SH2 domains, to other RTKs including the platelet-derived growth factor (PDGF) and epidermal growth factor (EGF) receptors [32]. In SHP-2, the engagement of the N-SH2 domain is required for its activation [33,34]. By inference, in our case, SHP-2 recruitment to tyrosine phosphorylated MuSK may allow for proper SHP-2 localization at the muscle membrane, and at sites where its phosphatase activity would be required in postsynaptic stabilization. Possible downstream targets of recruited SHP-2 could be MuSK itself, allowing fine-tuning of MuSK phosphorylation levels in a feedback mechanism [22] or SFKs. SHP-2 is known to interact [35] and positively regulate Src activity, either directly or through intermediate proteins such as PAG and Csk [26,35,36]. SHP-2 could also associate with and dephosphorylate the AChR as proposed earlier [37]; the AChR is known to undergo dephosphorylation in myotubes, since pervanadate treatment rapidly causes strong AChR β phosphorylation [23].

### The balance does the trick: upon lack of balance by SFKs, PTPs destabilize AChR clusters

We unraveled the mechanism by which PTPs such as SHP-2 act in stabilizing postsynaptic AChR clusters by testing their interplay with SFKs. We used two models: *src*^-/-^;*fyn*^-/- ^myotubes, and wild-type (C2C12) myotubes treated with the specific inhibitor PP2. In both situations, agrin induced normal AChR clusters, but upon agrin withdrawal these clusters disintegrated more rapidly than in the parallel controls. Importantly, in both *src*^-/-^;*fyn*^-/- ^myotubes and PP2-treated C2C12 myotubes, blocking PTPs with pervanadate after agrin induction restored stability of the pre-existing agrin-induced clusters. This stabilization was a specific process by several criteria: pervanadate did not alter levels of spontaneous clusters; the stability, not the formation of clusters was affected by having less SFKs; and when added together with agrin, pervanadate did not affect formation of AChR clusters in *src*^-/-^;*fyn*^-/- ^myotubes. Interestingly, *src*^-/-^;*fyn*^-/- ^myotubes were found to have substantially elevated levels of SHP-2 protein. The elevated amounts may be the result of changes in SHP-2 synthesis, turnover or degradation, and the amounts may be an attempt of *src*^-/-^;*fyn*^-/- ^myotubes to produce normal SFK activity, because SHP-2 is known to activate SFKs, at least in other cells [26,35,36]. Along the same lines, levels of the SFK member Yes are upregulated in *src*^-/-^;*fyn*^-/- ^myotubes [13].

Collectively, our data show that in the absence of normal SFK activity, PTPs no longer act to stabilize AChR clusters (as is normally the case). Rather, PTPs, when not counterbalanced by SFKs, destabilize the clusters, and blocking PTP activity restores cluster stability. In a related mechanism, agrin activates PTKs as well as PTPs in *Xenopus *muscle cells, and this allows formation of new AChR clusters but at the same time causes disassembly of pre-existing spontaneous AChR hotspots. Hotspot disassembly is blocked by pervanadate, implying that agrin-activated PTPs disassemble AChR clusters when they are not protected by local agrin-activated PTKs [22].

Our findings strengthen the proposal that it is the balance between PTKs such as SFKs and PTPs such as SHP-2, which controls AChR cluster stability. In *src*^-/-^;*fyn*^-/- ^myotubes, elevated SHP-2 levels could be causing the instability of AChR clusters. Using the inhibitor pervanadate on these myotubes could bring down the phosphatase activity, normalizing the PTP-PTK balance in the system, and allowing for more stable AChR clusters. On the other hand, in wild-type myotubes phosphatase inhibition by pervanadate reduces the phosphatase activity below normal levels, tipping the balance towards increased PTK activity, and leading to a destabilization of the system and consequently of AChR clusters.

The balance of SFKs and SHP-2 also plays *in vivo*: very similar to our present findings on SHP-2, it was recently shown, using the same electroporation technique, that a decrease or increase in SFK activity causes disassembly of postsynaptic pretzel-shaped AChR clusters and often displacement of subsynaptic myonuclei [11]. Thus interfering with SFK activity produces the same effect as reducing SHP-2 function at NMJs *in vivo*.

Our results describe a "phosphostat", which is required to keep AChR clusters optimally stable, but what could be the effector machinery that translates this phosphostat into postsynaptic stability? Most likely it is not just a classic simple mechanism where PTKs phosphorylate targets whereas PTPs dephosphorylate them. Rather, it appears to be a combination of common phosphorylation substrates and mutual control of activity of PTKs and PTPs. Since SHP-2 positively controls the activity of Src kinase [26], block of SHP-2 would result in less Src activity, leading to unstable AChR clusters. This may indeed be part of the situation in our C2C12 myotubes and at NMJs *in vivo*, where PTP block or SHP-2 knockdown destabilizes AChR clusters. On the other hand, AChR β subunit phosphorylation is a factor for efficient receptor clustering and cytoskeletal anchorage, and the AChR is dephosphorylated by PTPs, as shown by its overphosphorylation due to pervanadate treatment [23,38], also in our cultures (data not shown). With this in mind, blocking PTPs would be expected to lead to more stable AChR phosphorylation and thus to more stable clusters. However, the fact that pervanadate treatment increases AChR phosphorylation but destabilizes AChR clusters shows that phosphorylation of the receptor itself is not sufficient to keep clusters stable. Rather, other processes govern the stability of AChR aggregates, and SHP-2-mediated SFK activation governing activation of downstream substrates is one possibility *in vitro *and *in vivo*.

In the absence of SFKs, unbalanced PTP activity leads to dephosphorylation of AChRs, as shown in the agrin withdrawal phase in *src*^-/-^;*fyn*^-/- ^myotubes [11], and most likely of other downstream substrates such as cytoskeletal organizers. Accordingly, the strength of the overall cytoskeletal link of AChRs is reduced in *src*^-/-^;*fyn*^-/- ^myotubes [11]. From studies on non-muscle cells, cortactin, p190RhoGAP and WASp are known direct substrates of SFKs and involved, via direct or indirect action upon the Arp2/3 complex, in the dynamics of actin filaments [39,40]. Actin reorganization, in turn, and the action of Rac and Cdc42 (which can regulate cortactin and WASp, respectively) are important in clustering of AChRs [41-43]. Dephosphorylation of such regulators in the absence of SFKs could be a further reason for postsynaptic instability [11], and blocking PTPs could restore the activity of the regulators to normal operating levels, thereby stabilizing clusters.

**Figure 8:**
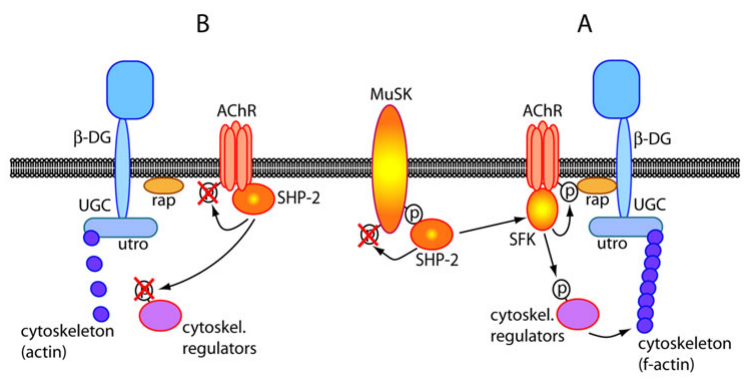
**Model for a balance between PTPs such as SHP-2 and kinases such as SFKs, which stabilizes postsynaptic AChR clusters**. Process A, stabilization: MuSK-bound and activated SHP-2 could activate SFKs, leading to AChR β phosphorylation, stable AChR-rapsyn (rap) interaction and phosphorylation of cytoskeletal regulators (e.g. actin-controlling proteins such as the SFK-substrates cortactin, p190RhoGAP or WASp). Process B, destabilization: SHP-2 may dephosphorylate AChR β and the cytoskeletal regulators. A balance between A and B keeps clusters intact. UGC, utrophin-glycoprotein complex: an array of proteins that stabilize the postsynaptic apparatus. Members of this complex interact with rapsyn (β-dystroglycan) and actin (utrophin).

These considerations lead to a model in which, in the stabilization phase of postsynaptic AChR clusters, PTPs such as SHP-2 associate with MuSK. Besides fine-tuning MuSK dephosphorylation, PTPs keep, indirectly or directly, SFKs activated (Figure 8, process A). This activity maintains AChR β phosphorylation, AChR-rapsyn interaction [11] and may maintain phosphorylation of critical downstream cytoskeletal regulators at operating levels. In parallel, PTPs such as SHP-2 dephosphorylate AChRs and the downstream regulators (Figure 8, process B). Under normal circumstances the balance of process A and B leads to a certain level of AChR and substrate phosphorylation, keeping clusters stable. Upon block of PTPs, SFK activity is compromised and clusters unstable. In the absence of SFKs, process B dominates, destabilizing clusters, but PTP block rescues stability.

Thus the effector pathway that operates between this PTP-PTK phosphostat and postsynaptic stabilization through AChR phosphorylation and cytoskeletal intermediates could be complex and awaits further investigation. Another level of complexity lies in the possible compensation between different PTPs or between PTP families, and this could explain why upon inactivation of the SHP-2 gene in muscle, no clear effect was seen at NMJs *in vivo *[44].

## Conclusion

In summary, our data show that PTPs such as SHP-2 stabilize postsynaptic AChR clusters and that the fine balance between PTPs and SFKs is a key aspect in this process. The data are the first, to our knowledge, to demonstrate a role for a tyrosine phosphatase in postsynaptic stabilization of a synapse in the nervous system *in vivo*. Previous reports concentrated on the role of PTPs in neurotransmitter receptor trafficking underlying synaptic plasticity [45], identified presynaptic PTP actions [46,47], or were limited to *in vitro *cultured neurons [48].

In our experiments, pharmacological inhibition of PTPs or knockdown of SHP-2 render AChR clusters less stable, whereas PTP inhibition restores the defective stability of clusters in myotubes lacking SFK activity. In addition, *src*^-/-^;*fyn*^-/- ^myotubes, which have unstable AChR clusters, show elevated levels of SHP-2 protein. Thus, under normal circumstances PTPs such as SHP-2 stabilize AChR clusters; but when these PTPs they are not balanced by SFKs, then they destabilize clusters.

While the crucial role of a balance between PTPs such as SHP-2 and kinases such as SFKs for AChR cluster maintenance is clear from the present study, the underlying cytoskeletal pathways and downstream targets await further investigation.

## Methods

### Expression of agrin and cell culture

The agrin used in this paper is a soluble neural agrin construct (C-Ag_12,4,8_) [49] and was produced in COS cells as previously described [50]. C-Ag_12,4,8 _lacks the N-terminal half of agrin including the laminin binding site but can interact with dystroglycan, activate MuSK and induce clustering of postsynaptic proteins in cultured myotubes, as full-length agrin does [51,52]. Reagents for cell culture were purchased from Invitrogen AG (Basel, Switzerland). C2C12 mouse muscle cells were propagated as myoblasts on 3.5 or 6 cm (Corning), and 10 or 15 cm plastic dishes (Nunc) in Dulbecco's modified Eagle's medium (DMEM) with 4.5 g/l D-glucose and pyruvate, supplemented with 20% fetal bovine serum, 0.5% chick embryo extract, 2 mM glutamine and penicillin/streptomycin. After reaching 90–100% confluence, cells were shifted to fusion medium containing DMEM, 5% horse serum and 2 mM glutamine and penicillin/streptomycin. Fusion of myoblasts to generate myotubes was evident after 1 day. By 2–3 days, contracting myotubes were usually observed, and cells were used for experiments [50]. *src*^-/-^;*fyn*^-/- ^myoblasts (clone DM15), and the corresponding wild-type myoblasts (SW10) were grown in 6 and 10 cm plastic plates (Nunc) in DM growth medium and switched to DM fusion medium to form myotubes as previously described [11,14].

### Inhibitors

Sodium pervanadate was prepared as previously described [23,53]. One part of 500 mM hydrogen peroxide was added to 50 parts of 10 mM sodium orthovanadate (Sigma) (pre-boiled at 100°C for 10 minutes) in modified Tyrodes solution (145 mM NaCl, 5 mM KCl, 5.5 mM glucose, 40 μM CaCl_2_, 1 mM MgCl_2_, and 10 mM HEPES pH 7.4). The mixture was shaken for 10 min at room temperature and diluted in cell culture fusion medium to defined concentrations immediately before use. Cultures were routinely treated with 20 μM pervanadate. Src-class kinase inhibitor PP2 [4-amino-5-(4-chlorophenyl)-7-(*t*-butyl)pyrazolo[3,4-d]pyrimidine] (Calbiochem) was diluted into cell culture medium to a final concentration of 10 μM [14,54].

### Antibodies

Antibodies against phosphotyrosine (PY20, 4G10); the AChR β subunit (mAb35); the conserved C-terminus of Src, Fyn, and Yes (*src*-CT); MuSK; rapsyn; and β-dystroglycan were all used as previously described [17,55,56]. Human anti-MuSK serum was obtained from Dr. Angela Vincent, Oxford, UK. It originates from myasthenia gravis patients who had high concentrations of anti-MuSK antibodies in their bloodstream. Experiments for Figure 3 were carried out with this serum and also with rabbit anti-MuSK antibodies [50], producing identical results. SHP-2 antibody (sc-7384; Santa Cruz Biotechnology Inc., CA, USA); Alexa Fluor^® ^488 anti-mouse and Alexa Fluor^® ^546 anti-rabbit (Molecular Probes, Eugene, OR, USA); rabbit polyclonal c-Myc antibody (sc-789; Santa Cruz Biotechnology Inc., CA, USA); and mouse monoclonal Flag M2 antibody (F3165; Sigma, Switzerland) were used as indicated by the suppliers.

### Assay for stability of AChR clusters

To study the effect of pervanadate on the stability of agrin-induced AChR clusters, C2C12 myotubes were treated with agrin (1 nM) for 6–8 hours to induce clustering, and were subsequently washed twice with fusion medium and maintained in fusion medium lacking agrin for 16 h, in the presence or absence of pervanadate (20 μM). The washing procedure was shown to be efficient in removing the vast majority of agrin from cells, one reason presumably being the lack of the laminin-binding site in C-Ag_12,4,8 _[7]. In controls, C2C12 myotubes were treated with agrin for 6–8 h followed by withdrawal of agrin for 16 h, myotubes were simply treated with agrin for 6–8 h, or cultures were left untreated. In *src*^-/-^;*fyn*^-/- ^and corresponding wild-type cells, following a 16 h treatment with agrin, cultures were washed twice with DM fusion medium and maintained in fusion medium for 5 hours, in the presence or absence of pervanadate. In controls, *src*^-/-^;*fyn*^-/- ^and corresponding wild-type cells were incubated with agrin for 16 h followed by withdrawal for 5 h, incubated with agrin for 16 h, or cultures were left untreated.

To study the requirement for both SFKs and PTPs for the stability of agrin-induced AChR clusters, cultures were treated with agrin for 6–8 hours. Src-class kinase inhibitor PP2 (10 μM) was added to cultures during the last 2 hours of agrin treatment [14,54]. Cultures were then washed twice with fusion medium followed by a 16 h incubation in fusion medium including PP2, or PP2 and pervanadate.

### AChR clustering assay and quantification of clusters

To study the effects of inhibitors and of SHP-2 shRNA on AChR cluster formation or stability, AChR clusters were visualized by incubating myotube cultures grown in 3.5 cm dishes with 100 nM tetramethylrhodamine-conjugated α-bungarotoxin (α-BTX) (Molecular Probes) in fusion medium for 1 hour at 37°C followed by fixation in 3% paraformaldehyde (PFA) in potassium phosphate buffer containing 4% sucrose for 15 minutes at room temperature, or in methanol for 7 minutes at -20°C [55]. Myotubes were examined at 400× magnification in both rhodamine and fluorescein channels with a fluorescence microscope (Axioskop II; Zeiss). Representative pictures (1344 × 1024 pixels) were taken and processed with a cooled digital camera (Orcacam; Hamamatsu) and SimplePCI software run on a Dell Dimension 8300 personal computer. The exposure times for individual channels were kept constant in all experiments.

AChR clusters were quantified using the NIH ImageJ 1.34 software. Images were opened in ImageJ; the scale was set to 3.8 pixels/μm for pictures taken at 400×, and to 1.9 pixels/μm for pictures taken at 200×. The threshold levels were set to 150–255, depending on the general background. Particles were analyzed, with the minimum particle size defined at 100 pixels. Data extracted from each picture included minimum and maximum particle size, mean particle size, mean particle area and number of particles. Particles >100 pixels approximated very closely to what would be considered as a cluster >10 μm length as judged by eye, and were thus taken as representative of a single AChR cluster. AChR clusters were quantified from approximately 15 random fields per experiment, and the mean ± SEM number of clusters per field was determined. We did several trial quantitations by both this automated method and by eye, and the outcome was very similar – hence our method reports very closely what can be seen by eye (A. A. Camilleri and C. Fuhrer, unpublished observations).

### Immunoprecipitations and immunoblot analysis

For immunoprecipitation of MuSK, cell lysates from cells grown on 10 cm plates were prepared as previously described [7,50], and MuSK precipitated using human anti-MuSK sera or rabbit MuSK antibodies, followed by protein A or G sepharose beads (GE Healthcare, Uppsala, Sweden), as previously described [7,50]. Following immunoprecipitations, all samples were loaded on sodium dodecyl sulfate (SDS)-polyacrylamide gels and probed using a mixture of phosphotyrosine antibodies 4G10 and PY20, and subsequently anti-MuSK or anti-SHP-2 antibodies (details above). Quantitations of immunoblots were done by scanning exposed films containing grey, nonsaturated signals with a computerized densitometer (HP Scanjet 5530) and using the NIH ImageJ 1.34 software. Experiments were repeated seven times, to obtain consistent results.

To analyze the effects of SHP-2 knockdown on the endogenous levels of SHP-2 and several other postsynaptic proteins, mature transfected myotubes were lysed, and equal amounts of proteins were loaded onto SDS gels. Following transfer to nitrocellulose paper, postsynaptic proteins were probed using specific antibodies against rapsyn, β-DG, MuSK, SHP-2, AChR β subunit and Src-CT.

### Expression constructs and shRNA

Myc-tagged wild-type MuSK expression construct, *MuSK-myc *(*pMuSK_myc*), was a gift of Prof. H.R. Brenner (Department of Physiology, University of Basel, Switzerland) [57]. Wild-type SHP-2 (obtained from Dr. J.L. Bixby (University of Miami School of Medicine, Miami, USA)) [58] was subcloned into *Bam/Not *of *pcDNAI-Flag *to obtain a *SHP-2-Flag *(*pcDNAI-SHP-2-Flag*) construct. The constructs were transfected into COS cells using Fugene6 (Roche, Basel, Switzerland) as described below.

*pSUPER.neo+gfp *vector (*pSUPER*) was purchased from OligoEngine (Seattle, USA). A shRNA (short-hairpin RNA) construct against murine SHP-2 (NCBI Accession number NM_011202) was generated by cloning the target sequence (5'-gaatacggggtcatgcgtgtt-3') into the *BglII/XhoI *sites of the *pSUPER *vector (adapted from [27]) according to the manufacturer's recommendations.

### Transfections

Transfections of constructs into C2C12 and COS cells were carried out using Fugene6 transfection reagent (Roche, Basel, Switzerland) according to the manufacturer's recommendations. Optimal transfection conditions were established by transfecting C2C12 myoblasts at 70–90% confluence. Two days later cells were induced to fuse by switching to fusion medium for 2 days. Transfected cells were observed by the expression of green fluorescence protein (GFP). Transfection efficiency on the myoblast stage was at 40–50%, and at the myotube stage (following fusion) was estimated at 80–100%. For co-transfection of COS cells with *MuSK-myc *and *SHP-2-Flag *constructs, equal quantities of DNA were transfected.

### ^125^I-α-bungarotoxin binding assay

To measure the surface level of AChRs following SHP-2 knockdown by shRNA, myotube cultures grown in 3.5 cm plates were incubated with ^125^I-α-BTX (Amersham Biosciences, Arlington Heights, IL) for 1 hour at 37°C. Cultures were washed twice with cold PBS supplemented with 1 mM Na orthovanadate and 50 mM NaF and lysed at 4°C in 1 ml lysis buffer [50] for 15 minutes. Cell lysate aliquots were taken for protein determination assays. Radioactivity from surface AChR-bound ^125^I-α-BTX was counted in an LKB Wallac 1282 CompuGamma counter (2 min counting mode). Non-specific binding was determined using 1 μM unlabelled α-BTX (1 hour pre-incubation and during radioactive labeling) [54]. Radioactive signals were robust (30000–100000 cpm), and the non-specific background was low (5–10%). Counts were normalized for the total protein content for each sample. AChR surface levels following SHP-2 knockdown by shRNA were plotted as percentages of *pSUPER *transfected controls.

### In vivo electroporation of soleus muscles, whole-mount preparation, and immunohistochemistry

Targeting of SHP-2 *in vivo *was achieved by electroporating the shRNA construct *pSUPER.shSHP2 *(2 or 4 μg/μl final concentration) into the soleus muscle of mice, together with green fluorescent protein (GFP) containing a nuclear localization signal (NLS-GFP; 2 μg/μl). In control mice, soleus muscles were electroporated with *pSUPER *vector construct (2 or 4 μg/μl) together with NLS-GFP (2 μg/μl). DNA constructs were pre-mixed and injected into the soleus muscle of adult C57BL/6 mice (8–11 weeks of age). After closing the wound, electrodes were mounted against the leg, and electroporation was performed as described previously [8] using an ECM 830 electroporation system (BTX, Holliston, MA). The procedure for the electroporation, dissection, fixation, and whole mount preparation of the muscle fibers was carried out as detailed previously [8,11]. Muscle fibers were stained with polyclonal anti-neurofilament and anti-synaptophysin antibodies followed by Cy5-conjugated goat anti-rabbit antibodies to visualize the nerve, as well as with rhodamine-α-BTX to detect AChRs, as previously described [8,11].

### Confocal imaging, image processing and quantitations

Confocal microscopy was used to analyze the mounted, fixed and stained muscle fibers. A Leica SP5 laser scanning microscope (Leica Microsystems, Wetzlar, Germany) running on a Hewlett-Packard Workstation xw6400, and a 20× oil-immersion lens, with settings specific for EGFP (green), rhodamine (red) and Cy5 (blue), were used. The three channels were detected sequentially, adjusting the laser power and detection windows individually for each channel, so as to exclude bleed-through. Confocal stacks were generated, and image processing, analysis, and three-dimensional reconstructions of confocal stacks was carried out using Imaris 5.0.1 software (Bitplane, Zurich, Switzerland) as previously described [8,11]. Assessment of NMJ disassembly and quantitation were done as detailed before [11]. Briefly, only endplates having GFP-positive synapse-associated nuclei were taken into consideration for quantitation, to make sure that NMJs corresponded to electroporated myofibers. Intact endplates were large, compact, pretzel-shaped structures of labelled AChRs, appearing brightly stained and continuous along their contours. Endplates were considered disassembled when the typical pretzel shape was no longer present, or endplates were crumpled and broken up into fragments. Quantitation of the number of intact or disassembled NMJs was done for each vector. A total of 25 endplates from three mice for the control *pSUPER *vector, and 15 endplates from five mice for the *pSUPER.shSHP2 *vector were analyzed.

## Authors' contributions

AAC and CF conceived the project and wrote the manuscript. AAC performed the studies of C2C12 and *src*^-/-^;*fyn*^-/- ^myotubes, RNA interference and *in vivo *electroporations, and performed all the statistical analyses. RW contributed to the studies on the MuSK and SHP-2 association, GS to the experiments with the *src*^-/-^;*fyn*^-/- ^myotube cultures, and MG to the cloning of shRNA constructs. SL and MAR contributed to the *in vivo *electroporations. RW and GS also contributed to the general concept. All authors read and approved the final manuscript.
